# Integrated Microfluidic Device for Drug Studies of Early *C. Elegans* Embryogenesis

**DOI:** 10.1002/advs.201700751

**Published:** 2018-03-08

**Authors:** Li Dong, Radek Jankele, Matteo Cornaglia, Thomas Lehnert, Pierre Gönczy, Martin A. M. Gijs

**Affiliations:** ^1^ Laboratory of Microsystems Ecole Polytechnique Fédérale de Lausanne (EPFL) Lausanne 1015 Switzerland; ^2^ Swiss Institute for Experimental Cancer Research (ISREC) School of Life Sciences Ecole Polytechnique Fédérale de Lausanne (EPFL) Lausanne 1015 Switzerland

**Keywords:** *C. elegans*, drug studies, embryogenesis, microfluidic devices

## Abstract

Small molecules inhibitors are powerful tools for studying multiple aspects of cell biology and stand at the forefront of drug discovery pipelines. However, in the early *Caenorhabditis elegans* (*C. elegans*) embryo, which is a powerful model system for cell and developmental biology, the use of small molecule inhibitors has been limited by the impermeability of the embryonic eggshell, the low‐throughput manual embryo isolation methods, and the lack of well‐controlled drug delivery protocols. This work reports a fully integrated microfluidic approach for studies of *C. elegans* early embryogenesis, including the possibility of testing small molecule inhibitors with increased throughput and versatility. The setup enables robust on‐chip extraction of embryos from gravid adult worms in a dedicated pillar array chamber by mechanical compression, followed by rapid fluidic transfer of embryos into an adjacent microtrap array. Parallel analysis of ≈100 embryos by high‐resolution time‐lapse imaging from the one‐cell stage zygote until hatching can be performed with this device. The implementation of versatile microfluidic protocols, in particular time‐controlled and reversible drug delivery to on‐chip immobilized embryos, demonstrates the potential of the device for biochemical and pharmacological assays.

The nematode *Caenorhabditis elegans* (*C. elegans*) is an attractive model organism, owing notably to its fast life cycle, genetic tractability, and large research community.^1^ About 40% of the ≈20 000 *C. elegans* protein coding genes are functional homologues of their human counterparts, including components of major signaling pathways that are conserved between the two species.^2^ Several human diseases have been modeled in *C. elegans*, allowing for high‐throughput drug screens, using either classical automated liquid handling protocols or microfluidics.^3^ Moreover, toxicity screens in adult *C. elegans* can provide a valuable entry point into understanding systemic responses to a variety of chemical compounds.^4^

To date, the majority of such studies utilized larval or adult worms, thus missing the unique opportunities provided by the early embryo, which is an excellent model system for analyzing fundamental cellular processes, such as cytoskeletal biophysics, cell cycle progression, and developmental regulation.^5^ Thanks to the optical transparency of the *C. elegans* embryo, perturbations of cellular processes can be observed with high spatial and temporal resolution by light and fluorescence microscopy.

Currently, the application of small molecules inhibitors to early *C. elegans* embryos is limited by the low rate of embryo harvesting and by partially controllable drug delivery. Typically, early embryos are obtained by manual dissection of adult hermaphrodite worms and are subsequently transferred onto agarose pads prepared on a microscope slide for imaging.^5^ Despite the fact that a trained person can isolate embryos relatively quickly, dissection sometimes must be repeated several times to capture the desired early embryonic stage, in particular in mutant or RNA interference (RNAi)‐mediated conditions. Moreover, the eggshell surrounding the embryo proper is impermeable to most small molecules, further complicating the use of *C. elegans* embryos for drug studies. Although RNAi‐mediated knockdown of the *perm‐1* gene has been used to circumvent this problem by rendering the eggshell permeable to small molecules with minimal deleterious effects,^6^ *perm‐1*(*RNAi*) embryos are fragile, making manual manipulation and drug application cumbersome. This issue can be mitigated by manually pushing embryos into a dedicated microwell array right after dissection.^6^ However, for the time being, there is no method specifically designed for convenient on‐chip handling and treatment of such fragile drug‐permeable early embryos of *C. elegans*.

Previously, our group introduced a microfluidic device for trapping nonpermeabilized embryos naturally laid by gravid *C. elegans* adults.^7^ This device allowed studying the different phases of embryogenesis after egg laying, which occurs typically after gastrulation has been initiated at the ≈30‐cell stage. Here, we describe the development of a novel integrated microfluidic approach enabling: (i) rapid and highly efficient extraction of embryos at earlier stages of development directly from gravid adults without perturbing embryo physiology, (ii) fluidic transfer and immobilization of single embryos in a microtrap array for high‐resolution imaging and analysis of development starting from the one‐cell stage, and (iii) precise handling and transport of small liquid quantities for controlled and versatile drug applications. We demonstrate the potential of the new device using time‐controlled delivery of the actin‐polymerization inhibitor Cytochalasin‐D (CD) to prevent cytokinesis in early embryos.^8^

Our device comprises two main functional polydimethylsiloxane (PDMS) components (**Figure** **1**a): an embryo extraction chamber including an array of partly compressible PDMS pillars featuring an innovative design for gentle embryo extraction (Figure 1b), and the linear trapping array for aligning immobilized individual embryos (Figure 1c). The extraction chamber (length 2 mm, width 2 mm, height 60 µm) is large enough to accommodate up to 20 *C. elegans* adult worms. It is bordered by specific on‐chip filter structures allowing for selective transfer of embryos excluding adult worms, carcasses, and debris (with a filter spacing of 60 µm on the In1 and Out1 side, and 30 µm on the In2 side and the side facing the trapping array, respectively). The principle underlying embryo extraction relies on their mechanical expulsion from the uterus of the worms via application of manual pressure pulses to the roof of the extraction chamber. This step is monitored under a stereo microscope. To prevent embryos from being smashed between the roof of the chamber and the coverslip during extraction, we developed an array of custom‐designed PDMS pillars, which securely maintains a minimal height of 30 µm when a pulse is applied on the roof of the chamber. These pillars feature two parts: a 40 µm high compressible and foldable crescent‐shaped structure and a 20 µm high, essentially incompressible, cylinder‐shaped pad on top of it (see Device Fabrication, Supporting Information). The pillar array can be either in a released or a compressed state (Figure 1d). In the released state, the pillar height is 60 µm, corresponding to the typical body centroid diameter of adult worms. By compressing the chamber, the foldable part of the pillars collapses onto the cylinder‐shaped pillar pad, eventually resulting in virtually incompressible structures. Considering the elastomeric properties of PDMS, we adjusted the geometrical parameters of the pillars to obtain a total height of ≈30 µm for the compressed structure. This arrangement is insensitive to variations in the pulses of applied manual pressure, such that embryos, whose centroid diameter is ≈30 µm, are shielded during the extraction procedure. Photographs of the pillar array in either state and of the full PDMS chip sealed to a glass coverslip are shown in Figure 1e–g, respectively.

**Figure 1:**
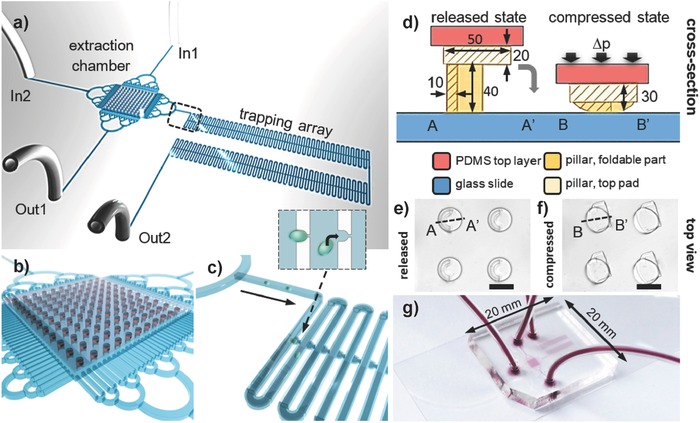
Microfluidic embryo harvesting and drug delivery chip. a) The integrated chip comprises a chamber with a reversibly compressible PDMS pillar array for extraction of embryos from gravid *C. elegans* worms, and an adjacent embryo trapping array. The device has two fluidic inlets (In1, In2) and two outlets (Out1, Out2). b) Schematic view of the extraction chamber with the pillar array and enclosing filter structures, and c) of embryo transfer into the trapping array. The inset shows a magnified view of the single‐embryo trapping process. d) Cross‐sectional views of a PDMS pillar (units are in µm), built from a foldable crescent‐shaped structure and a solid top pad, in its released (60 µm high) and compressed state (≈30 µm high), respectively. The foldable part faces the glass substrate. Pressure is applied on the chamber roof (a 5 mm thick PDMS top layer, not to scale in the figure). Top‐view images of a detail of the pillar array in the e) released and f) compressed state, respectively. Scale bar = 50 µm. g) Photograph of the PDMS‐on‐glass chip (20 mm × 20 mm) with fluidic connections. Microfluidic features are dyed in red.

The device is equipped with external flow control through two inlets (In) and two outlets (Out), via computer‐controlled syringe pumps that enable rapid and time‐controlled fluidic operations. First, a suspension of 10–15 gravid worms is introduced in the extraction chamber through the In1–Out1 fluidic path (**Figure** **2**a). Worms are blocked inside the extraction chamber by the bordering PDMS filter structures (Figure 2b). After mechanical expulsion of the embryos from the worms' uterus through the application of pressure, the embryos are dispersed in the chamber (Figure 2c). In general, almost all the embryos are released from the gravid worms after pressing three times on the chamber roof (Movie S1, Supporting Information). Thereafter, activation of flow along the In1–Out2 path transfers the embryos into the trapping array that is 40 µm in height, where they are immobilized by passive hydrodynamic action (Figure 2d). Each microtrap accommodates a single embryo (Figure 2e). An analysis of transfer and trapping rates is shown in Table S1 (Supporting Information). The yield of successful transfer of the released N2 wild‐type embryos was 91.0 ± 3.0% (*N* = 3), with the remaining embryos sticking to the walls of the extraction chamber. The trapping rate of transferred embryos was 95.7 ± 0.6% (*N* = 3). The total timespan from embryo extraction to imaging is 2–3 min. Thanks to the accurate geometric alignment of the microtraps, the locations of selected embryos can be readily recorded for subsequent imaging (Movie S1, Supporting Information). A motorized microscope stage is used to scan the array to find and mark positions of those traps containing embryos at the desired stage. The embryo array can receive up to 100 individual embryos at development stages that reflect the natural in utero distribution since most embryos are expulsed from the adult worms during extraction. Depending on the initial number of worms, the filling rate of the array is typically 60–90%. In this way, about ten wild‐type one‐cell stage embryos can be captured per experiment and selected for further analysis. Importantly, drug compounds can be simultaneously applied to all embryos trapped in the device via the In2–Out2 fluidic path (Figure 2f), and dynamic cellular processes can then be analyzed at high resolution. Laminar flow conditions and computer‐control allow for fast and reproducible fluidic exchange on‐chip (≈70 s at 100 nL s^−1^, Movie S2, Supporting Information), enabling accurate drug delivery protocols. A typical bright‐field image of a one‐cell wild‐type embryo in a microtrap is shown in Figure 2g.

**Figure 2:**
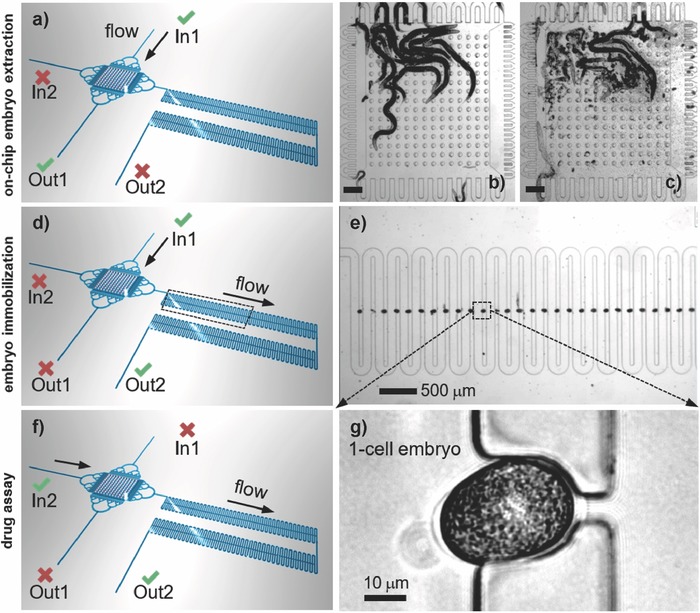
Protocol for on‐chip embryo extraction: a) Fluidic configuration for loading a suspension of adult worms into the extraction chamber (In1–Out1 open, In2–Out2 closed). b) Image of 14 adult worms confined in the chamber. The PDMS micropillar array is in the released state. c) Chamber after squeezing the worms by application of a pressure pulse on the chamber roof. A large number of extracted embryos populate the chamber. Scale bar = 200 µm. Microtrap array for embryo immobilization: d) Fluidic configuration for embryo transfer from the extraction chamber to the array (In2–Out1 closed, In1–Out2 open). e) Serpentine microchannel with microtraps for hydrodynamic immobilization of the embryos. The image shows a nearly completely filled portion of the array. Drug assay: f) Fluidic configuration for the perfusion of drug compounds on early embryos (In2–Out2 open, In1–Out1 closed). Drug compounds may be applied through the serpentine channel in a well‐controlled manner. g) A zoom on a one‐cell embryo securely positioned in a microfluidic trap adjacent to the serpentine channel (bright‐field, 50×/0.55 objective).

To test whether wild‐type *C. elegans* embryos develop normally in the microfluidic device, we monitored embryogenesis from the one‐cell stage all the way to hatching. For clarification, a graphical side view of a gravid adult *C. elegans* hermaphrodite is shown in **Figure** **3**. We found that our device enables the extraction and ex utero monitoring of early stages of development on‐chip up to hatching (using flow rates ≤100 nL s^−1^). Figure 3a–i is a sequence of representative bright‐field images of a wild‐type embryo captured in a microtrap after mechanical on‐chip extraction as described above. Several key events of embryogenesis are shown in Figure 3a–i. Figure 3a–c depicts the one‐ to four‐cell stage, for instance. At the end of the three‐fold stage (Figure 3h), the embryo starts moving inside the egg, indicating proper neuromuscular development. We found that hatching occurs ≈800 min after fertilization at 20 °C (Figure 3i), in line with established values on agar substrates.^9^ The on‐chip hatching rate of the trapped early embryos was 97.3 ± 2.5% (*N* = 3). A more detailed hatching rate analysis is provided in Table S2 (Supporting Information). Movie S3 (Supporting Information) monitors on‐chip embryogenesis from the one‐cell stage to hatching (corresponding to the snapshots shown in Figure 3a–i), and also shows a representative video of a microarray filled with embryos at different development stages. Proper embryo development and successive hatching events can be observed, indicating that our device and protocol provide adequate assay conditions. In particular, our chip allows secure ex utero manipulation of one‐cell embryos, which is most challenging because the eggshell is not fully established at this stage.^10^

**Figure 3:**
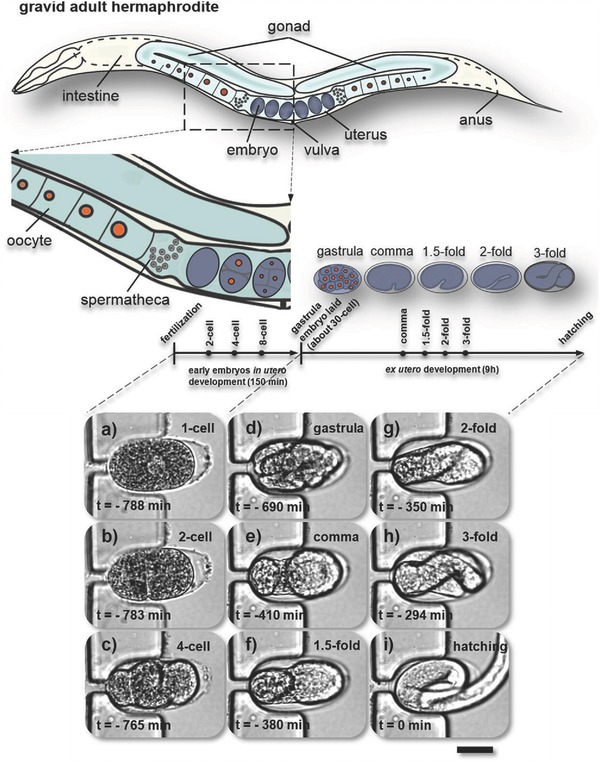
Study of complete *C. elegans* embryogenesis on‐chip. Schematic of anatomical structures of a gravid *C. elegans* hermaphrodite with two bilaterally symmetric gonad arms that are connected to a central uterus through the spermatheca. Embryos are shaded in blue‐gray. The embryonic development timeline shows several key events with distinguishable morphological changes during in utero (one‐cell to gastrula, ≈30 cells) and ex utero development (gastrula to hatching). a–i) Live imaging of normal on‐chip embryogenesis from the first division to larva hatching (*T* = 23 °C). Bright‐field images (50×/0.55 objective) of a microfluidic trap located in between two parallel sections of the serpentine channel in the trapping array (see Figure 2). a) An immobilized *C. elegans* embryo is shown in the one‐cell stage (a), and b–i) in subsequent main embryonic development phases. Note that this particular embryo was trapped with the anterior pole on the left. The moment of hatching defines *t* = 0 min. This sequence was taken over a timespan of ≈13 h (at the time points indicated in the figure), demonstrating reliable embryo positioning over prolonged periods. Scale bar = 20 µm.

The relatively big size and transparency of *C. elegans* embryos represent obvious advantages for analysis of mutant and RNAi conditions, and offers a potentially favorable setting for studying drug‐induced effects on cellular physiology and development. In particular, early embryogenesis has great potential for drug testing thanks to the relative simplicity and stereotypic spatiotemporal pattern of cell divisions, allowing for automated and quantitative analysis. During the first few cleavage divisions, six embryonic founder cells are generated (P_4_, D, C, E, MS, AB), whose descendants generate specific cell types and tissues (**Figure** **4**a).^9^ In order to test the potential of our newly developed device for performing drug assays, we set out to treat early *C. elegans* embryos transiently with CD, a well‐established actin polymerization inhibitor.^8^ Among other functions, the actin cytoskeleton is critical for cytokinesis, the final step of cell division, during which two daughter cells are produced from the mother cell by a contractile actin‐myosin ring that forms perpendicular to and in the middle of the mitotic spindle.^11^ Accordingly, CD can effectively block cytokinesis in mammalian cells and in *C. elegans* embryos.^8^, ^12^

**Figure 4:**
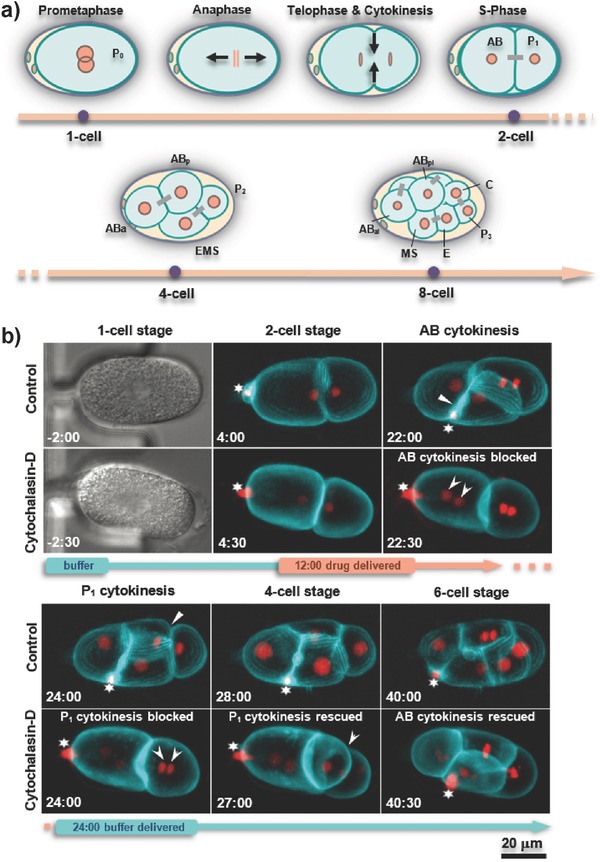
Early embryogenesis and on‐chip drug treatment. a) Schematic diagram of cleavage divisions until the eight‐cell stage. Daughter cells coming from a common mother cell are connected with a gray bar; black arrows indicate movement of chromosomes in anaphase and the constriction of the membrane during cytokinesis. b) Snapshots of development from time‐lapse recordings of permeable *perm‐1*(*RNAi*) embryos expressing GFP::PH on the membrane (cyan) and mCherry::histone‐H2B labeling chromatin (red) (63×/1.2 objective). Time is indicated in minutes, with *t* = 0 being metaphase in the one‐cell stage. The first and third rows of images show normal development of an untreated embryo immobilized in a microtrap (control), from prophase in the one‐cell stage until the six‐cell stage (white arrows indicate completed cytokinesis in AB and P_1_ cells, note that AB divides 2 min before P_1_). The second and fourth rows of images show time‐restricted drug delivery to a permeable embryo (the timing of drug delivery is indicated on the timeline by the orange section). Subsequent to drug delivery at the two‐cell stage (*t* = 12:00 min) for 12 min, cytokinesis was blocked in both AB and P_1_ cells, resulting in the presence of two nuclei after mitotic exit (marked by arrowheads at *t* = 22:30 and 24:00 min). After the drug was washed‐off, cytokinesis in P_1_ was rescued (*t* = 27:00 min). By contrast, AB underwent cytokinesis only after completion of the next S‐phase, demonstrating in both cases that CD was successfully washed off. The star indicates a polar body containing highly compacted DNA extruded during the female meiotic divisions, which is highly mobile within the eggshell.

For our study, we used drug‐permeable embryos extracted on‐chip from *perm‐1(RNAi*)‐treated gravid adult worms. The embryos were immobilized in the microfluidic trapping array and one‐cell embryos were selected for imaging prior to drug application. Whereas wild‐type adults always produce healthy early embryos in the trapping compartment (see Table S2, Supporting Information), we found that this was not always so in the case of *perm‐1(RNAi)*. The fragility of such embryos likely makes them more susceptible to the mechanical stress endured in particular during immobilization. Those *perm‐1(RNAi)* embryos that are compromised before drug exposure are not considered for further analysis (see Worm Strains Culture, Supporting Information, for further discussion of this point). In a typical experiment, 2–5 seemingly healthy *perm‐1(RNAi)* embryos can be found in the microarray, for which normal early development was observed (i.e., at least up to the 16‐cell stage). Embryos expressing GFP::PH (pleckstrin homology (PH) domain from rat Phospholipase‐C^13^) for visualizing cell membranes and mCherry::H2B (histone‐H2B) for marking the chromatin were analyzed by wide‐field fluorescence time‐lapse microscopy. Figure 4b demonstrates on‐chip drug treatment of an immobilized early embryo, and compares its early development to that of a control *perm‐1(RNAi)* embryo exposed to 0.1% dimethyl sulfoxide (DMSO), which exhibited a normal division pattern (Figure 4b, control row, and Movie S4, Supporting Information). In particular, it can be seen that the anterior AB cell divides ≈2 min before the smaller posterior P_1_ cell in the control condition (Figure 4b, control row, 22:00 and 24:00 min, respectively).^14^ For drug delivery, 10 × 10^−6^ m) CD was applied onto *perm‐1(RNAi)* embryos through the serpentine microchannel of the trapping array at a flow rate of 20 nL s^−1^ from *t* = 12 to 24 min in Figure 4b. During this time, failure of cytokinesis was observed in both AB and P_1_ blastomeres, leading to the presence of two nuclei per cell in each case after mitotic exit (Figure 4b, arrowheads in the CD rows at 22:30 and 24:00 min, respectively). Subsequently, the drug was readily washed out at *t* = 24 min within 3 min, and cytokinesis was rescued as a consequence (Figure 4b, arrowhead in the CD row at *t* = 27:00 min, and Movie S5, Supporting Information, respectively). Interestingly, we observed that whereas P_1_ could initiate cytokinesis in a delayed manner at this time point, this was not the case in AB, where cytokinesis occurred only after the subsequent S‐phase (*t* = 40:00 min), producing two instead of the usual four AB descendant cells. We observed similar reversible effects on cytokinesis in four embryos in three independent experiments.

In summary, we report the first fully integrated microfluidic device for drug studies on *C. elegans* early embryos. The new device enables direct on‐chip extraction of early embryos from gravid *C. elegans* hermaphrodites, and immobilization of released embryos, starting from the one‐cell stage, in a microtrap array for high‐resolution time‐lapse imaging. Microfluidic control enables accurate drug delivery protocols applied simultaneously to all embryos in the array. Transient drug exposure was demonstrated here by the arrest and then the rescue of cytokinesis in immobilized drug‐permeable *perm‐1(RNAi)* early embryos following time‐controlled delivery of CD. Further improvement of the device may include separate drug delivery channels and/or a second on‐chip array for simultaneous control assays (see Options for Parallel On‐Chip Control Experiments, Figures S1 and S2, Supporting Information), as well as automated and computer‐controlled application of the pressure pulses for embryo extraction. We anticipate that our approach will enable the implementation of early *C. elegans* embryo drug‐screening assays with increased throughput and versatility. For instance, dose–response assays could be performed on an integrated platform comprising a parallel arrangement of several embryo arrays combined with an on‐chip drug dilution mixer. We note that our chip could also be readily used for mutational or RNAi‐based screens in early embryos. In these regards, our device provides a novel and promising tool for future embryological research or pharmacological studies in the *C. elegans* model organism.

## Conflict of Interest

The authors declare no conflict of interest.

## Supporting information

Supplementary

Supplementary

Supplementary

Supplementary

Supplementary

Supplementary
