# Supplementary material for: Integrated Microfluidic Device for Drug Studies of Early C. Elegans Embryogenesis
Source: Adv Sci (Weinh). 2018 Mar 8;5(5):1700751. doi: 10.1002/advs.201700751 (PMC5980161; doi:10.1002/advs.201700751)
Supplement: Supplementary file 1 — Supplementary [file ADVS-5-1700751-s002.pdf]

Copyright WILEY-VCH Verlag GmbH & Co. KGaA, 69469 Weinheim, Germany, 2018.

ADVANCED SCIENCE
Open Access

Supporting Information

for *Adv. Sci.,* DOI: 10.1002/advs.201700751

Integrated Microfluidic Device for Drug Studies of Early *C. Elegans* Embryogenesis

*Li Dong, Radek Jankele, Matteo Cornaglia, Thomas Lehnert, Pierre Gönczy, and Martin A. M. Gijs**

Copyright WILEY-VCH Verlag GmbH & Co. KGaA, 69469 Weinheim, Germany, 2016.

Supporting Information

Integrated microfluidic device for drug studies of early *C. elegans* embryogenesis

*Li Dong, Radek Jankele, M. Cornaglia, Thomas Lehnert, Pierre Gönczy, and Martin A.M. Gijs**

Materials and methods

4-inch 550 μm thick Si and float glass wafers were provided by the EPFL Center of MicroNanoTechnology (EPFL-CMI). Polydimethylsiloxane (PDMS) Sylgard 184 was obtained from Dow Corning (Wiesbaden, Germany), negative photoresist (SU-8 3025) from micro resist technology GmbH (Berlin, Germany). Trimethylchlorosilane (TMCS) was purchased from Sigma-Aldrich (Buchs, Switzerland), 1 ml borosilicate H-TLL-PE syringes from Innovative Labor Systeme GmbH (Stützerbach, Germany), Microline ethylvinylacetate tubes with 0.51 mm inner diameter and 1.52 mm outer diameter from Fisher Scientific (Wohlen, Switzerland).

L-Broth bacterial culture medium was prepared by adding Bacto™ tryptone (10 g), Bacto™ yeast (5 g) and NaCl (5 g) in DI H_2O (1 L). L-Broth was sterilized by autoclaving. All chemicals used in L-Broth were purchased from Sigma-Aldrich (Buchs, Switzerland). Meiosis buffer was prepared by adding heat inactivated (30 min, 56°C) fetal calf serum (350 μl, FCS, Gibco BRL) to Shelton buffer (650 μl) [1]. Cytochalasin-D was obtained from AppliChem (Darmstadt, Germany). It was dissolved in dimethyl sulfoxide (~10 mM, 5 mg/ml, DMSO, Sigma-Aldrich) and stored at -20°C. The drug was then diluted in the meiosis buffer at 10 μM before use.

Worm strains culture

N2 wild-type *C. elegans* worms and worms expressing mCherry::H2B to mark chromatin and GFP::PH to mark cell membranes (strain GZ1326, generated by crossing strains OD57 and OD58 [2] from the *Caenorhabditis* Genetics Center) were used in this work. Strains were maintained at 25°C on nematode growth media (NGM) agar plates seeded with the auxotrophic *E. coli* strain OP50 according to standard methods [3]. In order to obtain embryos with a drug-permeable eggshell, the bacterial feeding strain expressing dsRNA against *perm-1* (T01H3.4) gene was retrieved from the Ahringer RNAi feeding library [4]. Bacteria were grown overnight at 37 °C in lysogeny broth medium (LB) with ampicillin (100 µg/ml) at 250 RPM. The culture was then diluted 1:10 in LB with ampicillin (100 µg/ml) and grown at 37 °C until reaching an optical density at 600 nm (OD600) between 0.5 - 0.7. Thereafter, the expression of dsRNA was induced with isopropyl-β-D-thiogalactopyranoside (1 mM, IPTG) and the culture was allowed to grow for one hour. The bacterial suspension was then spread on NGM agar plates (6 cm, ~350µl/plate) containing IPTG (0.1 mM). Plates were dried in a sterile hood for ~1 hour and left at room temperature for at least 24 hours before use. Subsequently, 20 to 30 young adults were transferred onto each plate and incubated at 20 °C for 12 - 15 hours. As reported previously [5], *perm-1*(*RNAi*) needs to be carefully calibrated, as insufficient inactivation results in embryos that are not permeable enough. By contrast, excess inactivation increases the fraction of early embryos that are fragile to a point that abnormal development ensues in all cells. Moreover, in some cases, we found that only one or several cells, in particular those adjacent to the trap edge, fail to develop properly, likely because of the embryo having experienced enhanced viscous shear stress during the immobilization process. *perm-1*(*RNAi*) inactivation conditions were chosen that maximized the fraction of embryos that developed entirely normally until drug addition, which were the only ones retained for further analysis.

Device fabrication

We used standard polydimethylsiloxane (PDMS) soft lithography techniques to fabricate the microfluidic chips (Figure 1). The master mold was made using a two-step process. First, patterns for all fluidic structures, in particular the trapping array and the foldable parts of the pillar array, were created on a silicon wafer by plasma etching (structure height 40 μm). Subsequently, a 20 μm thick SU-8 layer was spin-coated on the wafer and patterned to form the non-compressible pillar top pads, as well as to increase the total height of the main chamber and of the In1-Out1 fluidic path to 60 μm (Figure 1a). The mold surface was treated with TMCS for 60 min to facilitate PDMS demolding. PDMS was poured over the SU-8/Si mold to form a 5 mm thick layer and cured at 80 °C for 1 hour. PDMS chips were plasma-bonded onto 0.17 mm thin glass coverslips for observation.

Time-lapse microscopy

Figure 3: Brightfield images were obtained with a Carl Zeiss Axiovert 100 inverted microscope, a LD EC Epiplan-Neofluar 50×/0.55 BD DIC objective and a Hamamatsu Orca-ER High-Resolution Digital B/W CCD C4742-95 camera. Images were captured every 60 s (exposure time 2 ms).

Figure 4: Time-lapse dual DIC and fluorescence microscopy was performed with a Carl Zeiss Axio Observer.D1 inverted fluorescence microscope equipped with a motorized XY stage including a piezo Z-insert (both from Ludl Electronics Products, Ltd.), an C-Apochromat 63×/1.2 W DIC high-resolution water-immersion objective, an Andor Zyla 4.2 PLUS sCMOS B/W camera, and a SOLA LED light engine® (Lumencor, Inc.). One z-stack image (spacing 2 μm, 15 images per time point) was captured every 90 s in all three channels (DIC, GFP, RFP) using 50 and 75 ms exposure for GFP and mCherry, respectively (at 5% light output).

Movies were processed using ImageJ (background subtraction and maximal intensity projection of z-stacks) and Adobe Premiere Pro CC.

Table S1. Embryo transfer and trapping rate

'Transfer rate' is defined as the number of embryos (*t1*) entering the trapping array divided by the total number of released embryos (*re*) in the extraction chamber. 'Trapping rate' is defined as the number of immobilized embryos (*t2*) divided by the number of embryos (*t1*) entering the trapping array. The present on-chip embryo transfer and trapping rate analysis is based on three subsequent experiments (see also Movie S1). Nearly all worms loaded into the extraction chamber are compressed upon application of external pressure, except a few ones that are located too close to the border of the chamber.

assay	# worms		# embryos			transfer rate	trapping rate
	loaded	compressed	released (*re*)	transfered (*t1*)	trapped (*t2*)		
1	11	8	82	75	72	0.91	0.96
2	10	9	105	92	87	0.88	0.95
3	11	9	84	79	76	0.94	0.96

Table S2. Hatching rate for on-chip embryogenesis assays

On-chip hatching was analyzed for wild-type *C. elegans* early 1- to 8-cell stage embryos, as well as for embryos at later development stages (> 8 cells). Here, the 'early embryo hatching rate' is defined by the ratio of hatched embryos with respect to trapped early embryos (*h1*/*t1)*. The 'total embryo hatching rate' is defined by (*h1*+*h2*)/(*t1*+*t2*). Unfertilized embryos do not develop and are not included in the *t1* or *t2* count. This hatching rate analysis is based on three experiments (see also Figure 3 and Movie S3).

assay	1-cell to 8-cell (early embryos)		>8-cell		unfertilized		hatching rate	
	trapped (*t1*)	hatched (*h1*)	trapped (*t2*)	hatched (*h2*)	trapped	hatched	early embryos	total embryos
1	31	31	38	35	3	0	1.00	0.96
2	37	35	45	42	5	0	0.95	0.94

3	33	32	37	34	6	0	0.97	0.94

Options for parallel on-chip control experiments

In the present work, control experiments were performed on a fully separated chip in an independent manner (Fig. 4). Here, we show two options for partial (Fig. S1) or full integration of a parallel control experiment (Fig. S2).

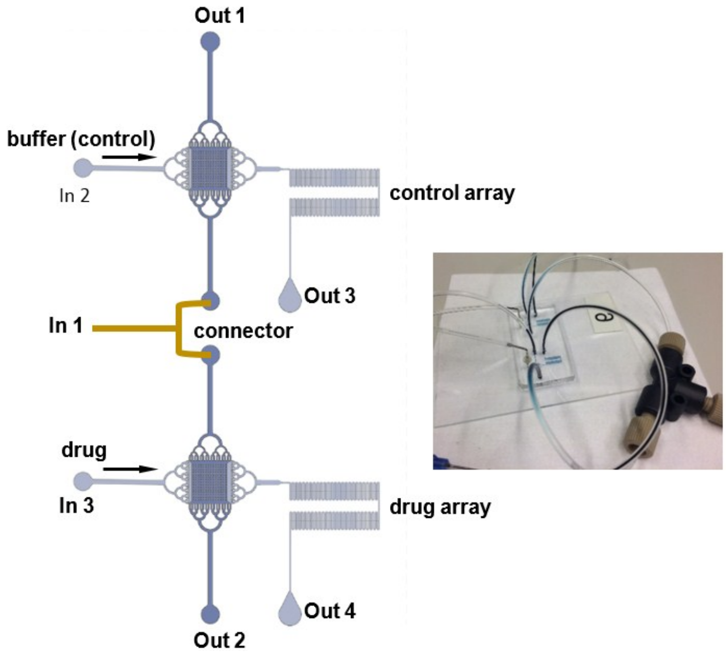

Figure S1. A drug application unit and a control unit are connected to a common inlet In1 by means of a T-connector (see photo). In1 is used to load worms simultaneously into the two units and to deliver buffer solution. For the assay, drug solution or buffer (control) are loaded through In2 or In3 of either chip unit, respectively.

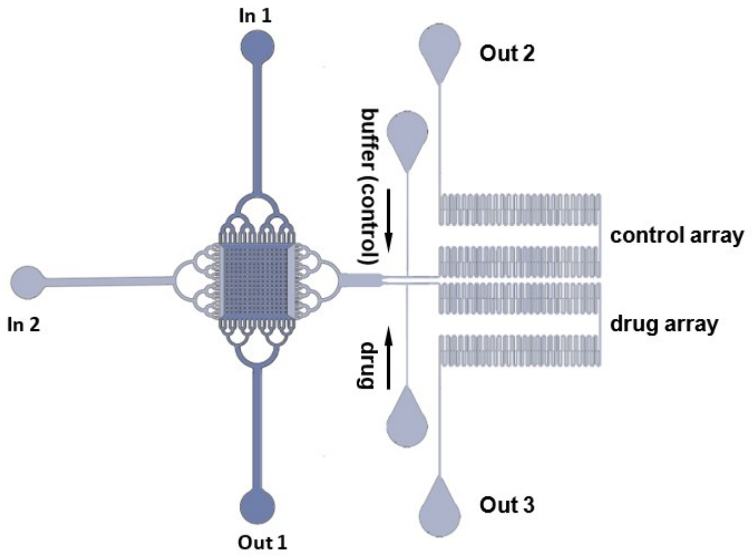

Figure S2. Fully integrated chip design with a single embryo extraction chamber and two parallel trapping arrays for the drug assay and the control, respectively. Drug solution or buffer is introduced at the indicated locations downstream with respect to the worm chamber. This configuration requires more careful fluidic control to avoid cross-contamination, *i.e.* drug leakage into the control channel. A simple solution would be to apply a very weak but continuous buffer flow through In2 during the assay. Another option is the integration of on-chip valves to isolate temporarily the two arrays.

References

[1] C. A. Shelton, and B. Bowerman, *Development* **1996**, *122*, 2043-2050.

[2] K. McNally, A. Audhya, K. Oegema, and F. J. McNally, *J. Cell. Biol.* **2006**, *175*, 881-91.

[3] S. Brenner, *Genetics* **1974**, *77*, 71-94.

[4] R. S. Kamath, A. G. Fraser, Y. Dong, G. Poulin, R. Durbin, M. Gotta, A. Kanapin, N. Le Bot, S. Moreno, M. Sohrmann, D. P. Welchman, P. Zipperlen, and J. Ahringer, *Nature,* **2003** *421*, 231-237.

[5] A. Carvalho, S. K. Olson, E. Gutierrez, K. Zhang, L. B. Noble, E. Zanin, A. Desai, A. Groisman, K. Oegema, *PloS one* **2011**, *6*, e24656.

Movie S1. On-chip extraction of embryos from gravid N2 wild-type worms, transfer of released embryos to the microtrap array and targeting a 1-cell embryo for drug assay. In this experiment, flow for transferring the embryos was applied manually by means of a 1 ml syringe. For all experiments involving fragile permeabilized embryos, a computer-controlled syringe pump for accurate flow control was used (using flow rates ≤100 nl/s, CETONI neMESYS syringe pump).

Movie S2. Demonstration of fast fluidic exchange in the embryo trapping microarray. Buffer and drug solutions are represented by yellow ink and clear water, respectively. The flow rate was 100 nl/s.

Movie S3. Full embryonic development on-chip from 1-cell stage until hatching as observed by brightfield microscopy (the inset shows a zoom on a single embryo trap). The background shows a video recording of the whole array. Most traps are filled with embryos, which progressively disappear after hatching.

Movie S4. Normal development on-chip of a *C. elegans* embryo in meiosis buffer + 0.1% DMSO from prometaphase in the 1-cell stage until anaphase in EMS at the 6-cell stage as observed by brightfield and fluorescence microscopies (time is indicated in minutes, with t = 0 min being at the first metaphase).

Movie S5. Embryogenesis upon on-chip delivery of the actin polymerization inhibitor Cytochalasin-D (10 μM in meiosis buffer + 0.1% DMSO) at the 2-cell stage (12:00 min) for a duration of 12 minutes as observed by brightfield and fluorescence microscopies (time is indicated in minutes, with t = 0 min being at the first metaphase).
